# Association between Adherence to Nutritional Guidelines, the Metabolic Syndrome and Adiposity Markers in a French Adult General Population

**DOI:** 10.1371/journal.pone.0076349

**Published:** 2013-10-04

**Authors:** Camille Lassale, Pilar Galan, Chantal Julia, Leopold Fezeu, Serge Hercberg, Emmanuelle Kesse-Guyot

**Affiliations:** 1 Université Paris 13, Sorbonne Paris Cité, Nutritional Epidemiology Research Unit, INSERM (U557), INRA (U1125), CNAM, Bobigny, France; 2 Public Health Department, Hôpital Avicenne, Université Paris 13, Bobigny, France; University of Sao Paulo, Brazil

## Abstract

**Introduction:**

Few studies have focused on the association between diet quality scores and the Metabolic Syndrome (MetS), a multi-component condition predictive of cardiovascular diseases (CVD) and death. The present study aims at investigating, in a cross-sectional design, the association between adherence to the French dietary guidelines through an a priori score – the French Nutrition and Health Program-Guideline Score (PNNS-GS) – and cardiovascular risk factors (CVRF) including the MetS and adiposity markers.

**Methods:**

7902 French adults participating in the NutriNet-Santé study (an on-going web-based cohort study) attended a clinical and biological examination between January 2011 and November 2012: a fasting blood sample was drawn, blood pressure and body composition (bio-impedance) were measured. Multivariate linear and logistic regression models were used to assess the association between PNNS-GS and CVRF or the MetS.

**Results:**

An increase of PNNS-GS was significantly negatively associated with waist circumference (WC), systolic and diastolic blood pressure (SBP and DBP) and serum triglycerides concentrations. From bottom to top quartile of PNNS-GS, SBP decreased from 129.9 to 128.8 mm Hg, DBP from 76.7 to 75.9 mm Hg, serum triglycerides concentrations from 110.8 to 104.6 mg/dL and WC from 94.8 to 90.1 cm for men and 81.3 to 78.9 cm for women. All adiposity markers (waist and hip circumference, % body fat, % trunk fat, % leg fat) were markedly reduced across quartiles of PNNS-GS and linearly. Individuals with a better PNNS-GS (quartile 4 vs quartile 1) were less likely to have the MetS (OR=0.71, 95% CI: 0.56-0.89).

**Conclusion:**

The negative association between a higher adherence to the French dietary guidelines and a number of CVRF, the MetS prevalence and regional adiposity supports the importance of promoting the PNNS dietary guidelines in the population for the prevention of cardiometabolic abnormalities and hence, cardiovascular diseases.

## Introduction

Cardiovascular disease (CVD) is a major public health burden in the western world and is likely to worsen with the increasing prevalence of obesity and type 2 diabetes [1]. Beside individual cardiovascular risk factors (CVRF), the Metabolic Syndrome (MetS) is a multi-component condition that includes a cluster of abnormalities, namely abdominal obesity, high blood pressure (BP), impaired fasting glucose/insulin resistance, high blood levels of triglycerides and low High Density Lipoprotein (HDL) cholesterol [2]. The MetS has been shown to be predictive of type 2 diabetes, atherosclerosis, CVD and mortality [3,4]. In this context, reducing the prevalence of cardiovascular risk factors and the MetS, by acting on diet and physical activity, is of major importance in the prevention of CVD.

A holistic approach of nutritional intake through dietary patterns and dietary scores can capture the combined effects of nutrients in the food matrix [5,6]. The role of the whole diet quality on adiposity and the MetS has been evaluated in observational studies [7,8] and several randomized controlled trials [9]. Adherence to a Mediterranean dietary pattern has been associated with lower body mass index (BMI) and waist circumference (WC) and with lower MetS prevalence and incidence according to recent meta-analyses and reviews [7-9]. Scarce other studies have evaluated the association between CVRF and adherence to dietary guidelines, using dietary scores such as the Dietary Guidelines for Americans Index (DGAI) [10,11] or the Healthy Eating Index (HEI-2005) [12,13]. In France, a score evaluating adherence to the French nutrition and health program (PNNS) dietary guidelines set up by the Ministry of Health in 2001 [14] has been developed, the Programme National Nutrition Santé Guideline Score (PNNS-GS) [15]. This score has shown negative association with the MetS and positive association with HDL cholesterol in a cross-sectional analysis on 1608 French adults but it was significant only among younger adults without medication, hence results were not generalizable to older adults [16]. A prospective analysis on a small sample (n=2763) of adults aged 45y and above from the SU. VI. MAX cohort has shown a predictive value of this score on MetS incidence after 6 years of follow-up (OR=0.91; 95% CI: 0.83-1.00) [17].

Beyond obesity, regional adiposity is a well-recognized independent CVRF [18] and it is of interest to study the links between dietary behaviours and adiposity markers. However, to our knowledge, only one study reported associations between the Mediterranean diet and accurate and specific measures of regional adiposity [19]. The aim of the present study was to evaluate in a large sample, in a cross-sectional design, the relationship between adherence to the French nutritional guidelines through the PNNS-GS and a comprehensive range of CVRF, the MetS and regional adiposity markers among French adults participating in the NutriNet-Santé cohort.

## Methods

### Study population and ethics statement

Participants were from the NutriNet-Santé study, an on-going web-based cohort study launched in France in May 2009 [20] aiming at investigating the associations between nutrition and health. Using a dedicated website, adult volunteers (aged >18 years) are followed for at least 10 years (recruitment still on-going). Electronic informed consent is obtained from all participants. All procedures were approved by the International Research Board of the French Institute for Health and Medical Research (IRB Inserm n° 0000388FWA00005831) and the French National Information and Citizen Freedom Committee “CNIL” (n° 908450 and n° 909216). The NutriNet-Santé study’s aims and methods have been described elsewhere [20]. Briefly, at inception, participants complete a set of questionnaires assessing demographic, socioeconomic and lifestyle factors, dietary intake, physical activity (PA), anthropometry and health status.

All participants in the NutriNet-Santé study are invited, on a voluntary basis, for a non-mandatory visit in one of the local centres specifically set up for biological sampling and clinical examination in each region (as of November 2012, 44 hospital-located centres were participating in the collection). Electronic and paper written informed consents are obtained from all subjects attending the visit. All procedures were approved by the “Consultation Committee for the Protection of Participants in Biomedical Research” (C09-42 on May 5^th^ 2010) and the CNIL (n° 1460707).

### Data collection

Apart from data collected at the clinical examination, all data used here were collected at inception of the cohort, through self-administered web-based questionnaires.

#### Dietary data and physical activity

Dietary intakes were assessed using three 24-hour records (24HR), randomly allocated over a two-week period, including two week days and one weekend day. The specific web-based tool for self-administered 24HR collection has shown high agreement with the reference method (interview with a dietician). For food groups, the median of Intraclass Correlation Coefficients (ICC) was 0.8 for men and 0.9 for women and for nutrients, the median of energy-adjusted Pearson correlation coefficients was 0.8 [21]. Participants reported all foods and beverages consumed at each eating occasion. Portion sizes were estimated with the help of photographs, derived from a previously validated picture booklet [22], that represent more than 250 generic foods, corresponding to more than 2000 specific food items, presented in three different portions sizes. Participants could also directly enter the quantity consumed in grams or volume, or use purchased units. Alcohol intake was calculated from the 24HR. The alcohol use frequency questionnaire was used if no consumption was reported in any of the three 24HR days. Consumption of fish and seafood per week was assessed by a specific frequency question as infrequently consumed food.

For each participant, daily mean food consumption was calculated from the three 24HR, weighted for the type of day (week or weekend day). Nutrient intakes were calculated using the ad-hoc NutriNet-Santé composition table including more than 2000 foods [23]. Identification of underreporting participants was based on the method developed by Black [24]. Participants detected as under-reporters were excluded from the analysis (10%). Only a few exclusions were made for over consumption because several controls were made to detect and, if applicable, correct implausibly high consumptions and intakes. Leisure time PA was assessed using the short form of the International Physical Activity Questionnaire (IPAQ), in the French language [25-27]. The metabolic equivalent (MET) measured in minutes per week was computed.

#### Clinical examination

During the visit, participants underwent a clinical examination as well as a fasting blood sampling. The clinical examination included measures of BP, weight, height, WC, Hip Circumference (HC) and bio-impedance measurements.

Height was measured once by a trained technician with a wall-mounted stadiometer without shoes to the nearest 0.5 cm [28]. Weight (to the nearest 0.1 kg) and body composition were measured once with a calibrated impedance body composition analyzer (BC-418MA, TANITA ©, Tokyo, Japan), with participants wearing indoor clothes, barefoot. Adiposity markers measured were the following: % body fat, trunk fat mass (kg) and % trunk fat, leg fat mass (kg) and % leg fat. Leg fat mass (kg) is the sum of fat mass in both legs, while % leg fat is the average % leg fat mass (leg fat mass/total leg mass) for both legs. The trunk to leg fat ratio (TLR) was computed as a measure of upper to lower body fat [19]. Waist circumference was measured as the circumference midway between the lower ribs and iliac crests, and hip circumference as the largest circumference between waist and thighs, both in a standing position and with an inelastic tape (nearest cm).

BP was measured three times after a 5-minute rest, for quality control, with a 1-minute lag between measurements, using an automatic sphygmomanometer Omron HEM-7015IT (Omron, Rosny-sous-Bois, France). The mean of the three measures was used for systolic and diastolic BP (SBP, DBP respectively).

#### Biomarkers

Blood samples were collected after a minimum of 6-h fast; all biochemical measurements were centralized at a single laboratory (IRSA, Tours, France). Fasting blood glucose (hexokinase on C 8000 automat, Abbott, Suresnes, France), total serum cholesterol (cholesterol oxidase C8000, Abbott), HDL cholesterol (direct accelerator C8000, Abbott), serum triglycerides (glycerol kinase C8000, Abbott) were measured and LDL cholesterol was calculated by Friedwald formula [29].

#### Covariate assessment

The following covariates were collected through self-administered questionnaires at baseline, which have been described elsewhere [30,31]. Education referred to the highest achieved diploma, occupation was either current job or most recent occupation for unemployed or retired individuals. Information was also collected on marital status, number of children, smoking status [31] and on use of medication for hypertension, diabetes or lipid-lowering medication, menopausal status (no, yes, current) and practice of restrictive diet [30].

### Data computation and Statistical analyses

#### PNNS-GS

The 15-point PNNS-GS is a validated a priori score reflecting the adherence to the official French nutritional recommendations which has been extensively described elsewhere [15]. Details on computation of this score are in Table S1. Briefly, it includes 13 components: eight refer to food-serving recommendations (fruit and vegetables; starchy foods; whole grain products; dairy products; meat, eggs and fish; fish and seafood; vegetable fat; water vs soda), four refer to moderation in consumption (added fat; salt; sweets; alcohol) and one component pertains to PA [14,15]. Points are deducted for overconsumption of salt (>12g/day), added sugars (>17.5% of energy intake), or when energy intake exceeds the needed energy level by more than 5%. Each component cut-off was that of the threshold defined by the PNNS public health objectives when available [14] otherwise they were established according to the French Recommended Dietary Allowances [32].

#### MetS

The MetS status was defined using the recent interim consensus statement [2] as having at least three of the following criteria: abdominal obesity (waist circumference ≥94 cm for men and ≥80 cm for women), high BP (SBP/DBP ≥130/85 mm Hg or antihypertensive medication), hypertriglyceridemia (≥150 mg/dL or antihypertriglyceridemia medication), low HDL-cholesterolemia (<40 mg/dL for men or <50mg/dL for women) and hyperglycaemia (fasting blood glucose ≥100 mg/dL or antidiabetic medication).

#### Anthropometrics

BMI was defined as weight divided by the square of height in meters (kg/m^2^). BMI categories of the WHO were used to define underweight (BMI<18.5), normal (18.5-24.9), overweight (25-29.9) and obese (≥30) subjects [33].

Waist to hip ratio (WHR) was calculated (WC/HC) as well as waist-to-height ratio (WHtR) as this index has gained popularity to predict cardiovascular risk [34].

#### Statistical Analysis

Characteristics of participants (socioeconomic and anthropometric) were compared between the study sample and the NutriNet-Santé cohort with available data for computation of PNNS-GS, by Student t-tests and chi square tests as appropriate. Quartiles of PNNS-GS were defined for the entire sample and for men and women separately.

To improve normality, all continuous variables were log-transformed. Effect modifications by gender were explored for each analysis.

We used multivariate linear regression to estimate adjusted geometric means of CVRF across quartiles of PNNS-GS. Contrast tests were conducted to detect presence of a linear trend. After assumptions for the application of linear regression were checked, multivariate linear regression models were also used to estimate change in outcome for the increase of 1 point of PNNS-GS.

For comparison purpose between outcomes, z-score of each health index was calculated. We employed exponentiation so that each coefficient could be interpreted as the percent change in z-score for 1 point increase in PNNS-GS [35]. All models were adjusted for the following covariates: sex, age (years), energy intake (kcal/day), season of completion of 24HR (spring, summer, fall, winter), time lag between inception and clinical visit (months), current dieting (yes/no), tobacco smoking (never, former, current smoker), occupation (never employed, farmers or self-employed, manual workers, blue-collar workers, managerial staff), education (up to high school, some college, university graduate) and treatment for the specific outcome (eg, antihypertensive drug when the outcome is SBD or DBP). Finally, BMI was also used as a covariate for analyses of all CVRF, except for WC. Analyses of WC were adjusted for body height.

The same analyses were carried out for adiposity markers stratified by sex, since the relation between diet and adiposity is likely to be sex-specific [36] and a significant interaction between sex and PNNS-GS was found for most of the adiposity markers. Covariates were the same as previously described, except BMI to avoid over adjustment. Furthermore, the following outcomes were also adjusted for body height: WC, HC, trunk and leg fat (kg). Models for women were further adjusted for menopausal status.

Logistic regression models were used to assess likelihood of having increased risk for MetS according to the level of adherence to dietary guidelines (PNNS-GS taken in quartiles and continuous). A first model was adjusted for the aforementioned covariates without BMI. A second model was further adjusted for BMI to evaluate the association of PNNS-GS with MetS beyond adiposity.

To further investigate the association of each recommendation with MetS and CVRF, a binary variable “recommendation reached” was created for each component, with a value of 1 if the score was ≥ 1 (0.8 for alcohol). OR of having MetS was estimated using the aforementioned models for each component of the PNNS-GS, further adjusted for the total PNNS-GS without the specific component and multivariate linear regression coefficients were estimated for each CVRF.

## Results

As of November 2012, out of 12 288 adult subjects of the NutriNet-Santé study who attended the visit between January 2011 and November 2012, 8745 had complete data to compute PNNS‑GS (dietary data from three 24HR, frequency of alcohol and of fish consumption, physical activity), while 7902 had complete data for CVRF needed to assess presence of MetS. At the same date, 63 378 adults with data available for computation of PNNS-GS were included in the NutriNet-Santé study (Table 1).

**Table 1:** Socioeconomic and lifestyle characteristics of the participants, NutriNet-Santé study, France, 2012.

	Men n=2 264		Women n=5 638			All n=7 902		Cohort n=63378		
	Mean	SD	Mean	SD	*P*-value^a^	Mean	SD	Mean	SD	*P*-value^b^
PNNS-GS	9.3	1.9	9.4	2.0	0.01	9.4	1.9	8.9	2.0	<.0001
Age (y)	54.5	13.6	49.3	13.3	<.0001	50.8	13.6	42.6	14.3	<.0001
	n	%	n	%		n	%	n	%	
% Women	-		-			71.4		77.6		<.0001
**Tobacco smoking**					<.0001					<.0001
Current smoker	240	10.6	617	10.9		857	10.8	10055	15.9	
Former smoker	1165	51.5	2064	36.6		3229	40.9	21575	34.0	
Never smoker	859	37.9	2957	52.5		3816	48.3	31746	50.1	
**On a weight loss diet**	567	25.0	3017	53.5		3584	45.4	30470	48.1	<.0001
**Living with a partner**	1780	78.6	3902	69.2	<.0001	5682	71.9	45891	72.4	0.32
**Occupation**					<.0001					<.0001
Never employed	26	1.2	142	2.5		168	2.1	3241	5.1	
Self-employed. farmers	92	4.1	134	2.4		226	2.9	2001	3.2	
Managerial staff	1354	59.8	2120	37.6		3474	44.0	21963	34.7	
Manual workers	48	2.1	44	0.8		92	1.2	1517	2.4	
Blue-collar workers	744	32.9	3198	56.7		3942	49.9	34654	54.7	
**Education ^c^**					0.50					<.0001
Up to high school	807	36.1	1764	32.1		2571	33.3	22071	35.6	
Some college	502	22.5	1752	31.9		2254	29.2	18666	30.1	
University graduate	926	41.4	1979	36.0		2905	37.6	21319	34.4	
**Season of 24HR**					0.38					<.0001
Spring	1199	53.0	3092	54.9		4291	54.3	36985	58.4	
Summer	375	16.6	970	17.2		1345	17.0	10324	16.3	
Fall	320	14.1	763	13.5		1083	13.7	8173	12.9	
Winter	370	16.3	813	14.4		1183	15.0	7896	12.5	
**Physical activity**					<.0001					<.0001
Low (<30 min/day)	379	16.7	1304	23.1		1683	21.3	16908	26.7	
Medium (30-<60 min/day)	500	22.1	1404	24.9		1904	24.1	15392	24.3	
High (>=60 min/day)	1385	61.2	2930	52.0		4315	54.6	31078	49.0	

Abbreviations: PNNS-GS, Programme National Nutrition Santé Guideline Score; SD, standard deviation

a P-value for difference between men and women. P-values for Student t-test (continuous variables) or Mantel-Haenzsel chi-square test (categorical variables).

b P-value for difference between study sample and total cohort. P-values for t-test (continuous variables) or Mantel-Haenszel chi-square test (categorical variables).

c Reduced sample size due to missing values

### Description of the participants

Compared with the NutriNet-Santé participants with data for PNNS-GS computation, subjects in our analysis sample were significantly older (50.8±13.6 vs 42.6 ± 14.3 y), had higher levels of physical activity, a higher PNNS-GS (9.4 ± 1.9 vs 8.9 ± 2.0), were less often smokers, were more often of urban setting, of high educational level and occupied a managerial position (all *p*-values<0.0001).

The study sample comprised 71% of women (vs 77.6% in the global sample of the NutriNet cohort). Compared to men, women were younger, had a slightly better PNNS-GS, were more often never smokers, were twice as likely as men to be on a diet, had lower physical activity levels and occupied less often a managerial position (all *p*-values<0.0001).

As described in Table 2, women were twice less likely to be overweight (18.6% vs 35.7%, p<0.0001) and to have MetS (9.5% vs 18.9%, p<0.0001), had lower BP, serum triglycerides and fasting blood glucose than men. Regarding adiposity markers, women had lower WHR, WHtR and TLR but higher % body fat.

**Table 2:** Cardiovascular risk factors among adults participating in the clinical and biological sample collection, NutriNet-Santé study, France, 2012.

	**Men n=2264**			**Women n=5638**				**All n=7902**		
	**n**	**%**		**n**	**%**			**n**	**%**	
**BMI category**							<.0001			
Underweight (<18.5)	19	0.84		229	4.06			248	3.14	
Normal (18.5-24.9)	1259	55.6		3929	59.7			5188	65.65	
Overweight (25-29.9)	809	35.7		1047	18.6			1856	23.5	
Obese (≥30)	177	7.8		433	7.7			610	7.7	
	**Mean**	**SD**	**n**	**Mean**	**SD**	**n**	**P-value ^a^**	**Mean**	**SD**	**n**
Time lag between dietary data collection and visit (months)	21.7	10.9		20.6	10.8		<.0001	20.9	10.9	
BMI (kg/m^2^)	25.4	3.7		23.9	4.4		<.0001	24.3	4.3	
SBP (mmHg)	133.5	15.7		121.9	15.5		<.0001	125.2	16.4	
DBP (mmHg)	78.6	9.6		74.6	9.2		<.0001	75.7	9.5	
Total Serum Cholesterol (mg/dl)	202.8	36.9		213.4	38.1		<.0001	210.4	38.1	
HDL-cholesterol (mg/dl)	55.2	12.1		65.8	13.7		<.0001	62.8	14.1	
LDL-cholesterol (mg/dl)	124.9	36.5		129.7	34.1		<.0001	128.3	34.9	
Blood triglycerides (mg/dl)	108.1	58.6		87.7	41.6		<.0001	93.5	48.0	
Fasting blood glucose (mg/dl)	93.4	12.8		87.7	10.0		<.0001	89.7	11.1	
Waist circumference (cm)	91.2	11.2		79.9	11.4		<.0001	83.1	12.4	
Hip circumference (cm)	98.4	6.9	2261	98.8	9.2	5636	0.01	98.7	8.6	7897
Waist to Hip ratio	0.93	0.07	2261	0.81	0.07	5636	<.0001	0.84	0.09	7897
Waist to Height ratio	0.52	0.07		0.49	0.07		<.0001	0.50	0.07	
% body fat	20.1	6.7	2235	29.7	7.7	5590	<.0001	27.0	8.6	7826
Trunk fat mass (kg)	9.8	4.9	2235	9.4	4.7	5590		9.5	4.8	7826
% trunk fat	21.4	8.0	2235	26.0	9.0	5590	<.0001	24.7	9.0	7826
Leg fat mass (kg)	4.7	2.1		8.3	2.9			7.3	3.2	7826
% leg fat	18.4	5.4	2235	35.8	6.1	5590	<.0001	30.8	9.9	7826
Trunk to leg ratio	2.06	0.53	2235	1.08	0.28	5590	<.0001	1.36	0.57	7826
	**n**	**%**		**n**	**%**			**n**	**%**	
**Metabolic Syndrome**	427	18.9		536	9.5		<.0001	963	12.2	
High triglycerides (≥150 mg/dL)or treatment	388	17.1		394	7.0		<.0001	782	9.9	
High BP (SBP ≥130 and/or DBP ≥85 mm Hg) or treatment	1381	61.0		1789	31.7		<.0001	3170	40.1	
High fasting blood glucose (≥100mg/dL) or treatment	465	20.5		527	9.4		<.0001	992	12.6	
Low HDL cholesterol (<40mg/dL males/<50mg/dL females)	173	7.6		595	10.6		<.0001	768	9.7	
Elevated WC (≥94cm for men, ≥80cm for women)	838	37.0		2467	43.8		<.0001	3305	41.8	
Treatment for hypercholesterolemia	287	12.7		340	6.0		<.0001	627	7.9	
Treatment for hypertriglyceridemia	47	2.1		25	0.4		<.0001	72	0.9	
Treatment for hypertension	380	16.8		481	8.6		<.0001	861	10.9	
Treatment for type 2 diabetes	59	2.6		38	0.7		<.0001	97	1.2	

Abbreviations: BMI, body mass index; SBP, systolic blood pressure; DBP, diastolic blood pressure; HDL, high density lipoprotein; LDL, low density lipoprotein; WC, waist circumference.

a P-value for difference between men and women. P-values for t-test (continuous variables) or Mantel-Haenszel chi-square test (BMI categories).

### Association between PNNS-GS and CVRF

SBP, DBP, triglycerides and WC significantly decreased across quartiles of PNNS-GS (Table 3). No significant trend was found either for serum cholesterol (total, HDL, LDL) or for fasting blood glucose. A 1-point increase of PNNS-GS was associated with a significant reduction in DBP (-1.38% of z-score, *P*=0.01), triglycerides (-2.16%, *P*<0.0001) and WC (-4.61%, *P*<0.0001).

**Table 3:** Cardiovascular risk factors according to adherence to the PNNS nutritional guidelines, NutriNet-Santé study, France, 2012.

	**Quartiles of PNNS-GS**							
**Variable**	**Q1 ^a^**	**Q2 ^a^**	**Q3 ^a^**	**Q4 ^a^**	**P ^b^**	**β % ^c^**	**95% CI**	
**PNNS-GS range**	**≤8.11**	**[8.11-9.50]**	**[9.50-10.79]**	**[10.79-14.80]**				
**Energy intake (kcal/day)^d^**	2118.4 ± 11.0	1937.6 ± 11.0	1849.0 ± 11.5	1812.1 ± 10.7	<.0001			
SBP (mm Hg)	129.9 (128.7-131.1)	129.0 (127.9-130.2)	129.3 (128.1-130.5)	128.8 (127.7-130.0)	<0.05	-0.58	-1.65	0.51
DBP (mm Hg)	76.7 (76.0-77.4)	76.4 (75.7-77.2)	76.3 (75.6-77.1)	75.9 (75.2-76.7)	0.01	-1.38	-2.53	-0.22
Total serum cholesterol (mg/dL)	197.7 (194.8-200.6)	199.3 (196.4-202.2)	198.7 (195.8-201.6)	198.2 (195.3-201.1)	0.79	-0.39	-1.55	0.77
HDL cholesterol (mg/dL)	57.6 (56.6-58.5)	58.2 (57.2-59.2)	58.1 (57.1-59.1)	58.2 (57.2-59.2)	0.13	0.04	-1.10	1.19
LDL cholesterol (mg/dL)	116.3 (113.8-118.8)	118.0 (115.5-120.6)	117.4 (114.9-120.0)	117.3 (114.8-119.9)	0.41	0.04	-1.12	1.23
Serum triglycerides (mg/dL)	110.8 (105.2-116.7)	107.4 (101.9-113.1)	107.7 (102.2-113.4)	104.6 (99.3-110.1)	<.0001	-2.16	-3.27	-1.03
Fasting blood glucose (mg/dL)	98.5 (97.3-99.7)	98.3 (97.1-99.5)	98.3 (97.1-99.6)	97.9 (96.7-99.2)	0.13	-0.04	-1.15	1.09
WC (cm)	86.6 (85.8-87.4)	85.6 (84.8-86.4)	84.8 (84.0-85.6)	83.6 (82.8-84.3)	<.0001	-4.61	-5.59	-3.62

Abbreviations: PNNS-GS, Programme National Nutrition Santé-Guideline Score; Q, quartile; 95% CI, 95% Confidence Interval; SBP, systolic blood pressure; DBP, diastolic blood pressure; BMI, body mass index; HDL, high density lipoprotein; LDL, low density lipoprotein,^a^ Values are geometric means (95% confidence interval) adjusted for gender, age, energy intake, tobacco smoking, current diet practice season of dietary data collection, occupational status, educational level, treatment for the specific outcome and BMI except for waist circumference that was adjusted for height.

b P-value for linear trend across quartiles using contrast test

c Multivariate linear regression models provide regression coefficients (β) for the difference in z-score of log-transformed variables for each 1 point increase in PNNS-GS. We used exponentiation so that each coefficient is interpreted as the percent change in expected z-score of variable for the increase of 1 point in PNNS-GS.

d Values are unadjusted means ± SD

Even though no significant association was found between PNNS-GS and cholesterol and blood glucose, every CVRF showed significant association with at least one individual dietary recommendation (Table S2).

### Association between PNNS-GS and adiposity markers

All adiposity markers displayed a linear decrease across quartiles of PNNS-GS, for both men and women (all *p*-values for linear trend <0.0001) (Table 4). For men, a dramatic fall in % body fat and in % trunk fat was observed between Q1 and Q4, from 21.2% to 17.7% and from 22.3% to 17.8% respectively. However for most markers for men, values were very similar between Q2 and Q3. As also reflected by the regression coefficients, the PNNS-GS was more strongly associated with adiposity markers in men than in women, e.g. for % trunk fat -9.11% (95% CI: –11.00% to -7.22%) vs -3.47% (95% CI: –4.79% to -2.15%).

**Table 4:** Adiposity markers according to adherence to nutritional guidelines (quartiles of PNNS-GS), men and women adults, NutriNet-Santé study, France, 2012.

	**Q1 ^a^**	**Q2 ^a^**	**Q3 ^a^**	**Q4 ^a^**	***P*^b^**	**n**	**β % ^c^**	**95% CI**	
**Men n=2 264**									
**PNNS-GS range**	**≤8.05**	**[8.05-9.30]**	**[9.30-10.70]**	**>10.70**					
BMI kg/m^2^	26.4 (25.9-26.9)	25.7 (25.2-26.2)	25.7 (25.2-26.2)	25.2 (24.7-25.7)	<.0001	2264	-6.49	-8.40	-4.54
WC (cm)^d^	94.8 (93.3-96.2)	92.5 (91.1-93.9)	92.1 (90.7-93.5)	90.1 (88.8-91.5)	<.0001	2264	-8.37	-10.15	-6.55
HC (cm)^d^	100.4 (99.5-101.3)	99.0 (98.1-99.9)	99.2 (98.3-100.1)	98.1 (97.3-99.0)	<.0001	2261	-6.57	-8.47	-4.63
WHR	0.94 (0.94-0.95)	0.93 (0.93-0.94)	0.93 (0.92-0.94)	0.92 (0.91-0.93)	<.0001	2261	-7.41	-9.19	-5.59
WHtR	0.54 (0.54-0.55)	0.53 (0.52-0.54)	0.53 (0.52-0.54)	0.52 (0.51-0.52)	<.0001	2264	-8.26	-10.00	-6.47
% body fat	21.2 (20.1-22.4)	19.7 (18.7-20.8)	19.7 (18.6-20.7)	17.7 (16.8-18.7)	<.0001	2235	-8.60	-10.40	-6.76
trunk fat mass (kg)^d^	10.2 (9.4-11.0)	9.1 (8.4-9.9)	9.0 (8.3-9.8)	7.7 (7.1-8.4)	<.0001	2235	-7.27	-9.12	-5.38
% trunk fat	22.3 (20.9-23.9)	20.5 (19.2-21.9)	20.3 (19.0-21.7)	17.8 (16.6-19.0)	<.0001	2235	-9.11	-11.00	-7.22
leg fat mass (kg)^d^	4.99 (4.73-5.27)	4.58 (4.34-4.83)	4.61 (4.37-4.87)	4.26 (4.03-4.49)	<.0001	2235	-9.05	-10.88	-7.18
% leg fat	19.5 (18.8-20.3)	18.4 (17.7-19.2)	18.6 (17.9-19.3)	17.4 (16.8-18.1)	<.0001	2235	-6.81	-8.56	-5.02
TLR	2.04 (1.95-2.13)	1.98 (1.90-2.07)	1.95 (1.86-2.04)	1.81 (1.73-1.89)	<.0001	2235	-8.11	-10.10	-6.05
**Women n=5 638**									
**PNNS-GS range**	**≤8.17**	**[8.17-9.52]**	**[9.52-10.80]**	**>10.8**					
BMI kg/m^2^	24.0 (23.5-24.4)	23.9 (23.5-24.3)	23.7 (23.3-24.1)	23.3 (22.8-23.7)	<.0001	5639	-2.73	-3.99	-1.45
WC (cm)^d^	81.3 (80.2-82.5)	80.8 (79.7-81.9)	79.9 (78.8-81.0)	78.9 (77.8-80.0)	<.0001	5639	-3.96	-5.21	-2.69
HC (cm)^d^	99.3 (98.4-100.2)	98.9 (98.0-99.8)	98.5 (97.5-99.4)	97.6 (96.6-98.5)	<.0001	5636	-3.28	-4.57	-1.97
WHR	0.82 (0.81-0.83)	0.82 (0.81-0.82)	0.81 (0.80-0.82)	0.81 (0.80-0.82)	<.0001	5636	-2.92	-4.22	-1.60
WHtR	0.50 (0.49-0.51)	0.50 (0.49-0.50)	0.49 (0.48-0.50)	0.49 (0.48-0.49)	<.0001	5639	-4.09	-5.32	-2.85
% body fat	29.5 (28.7-30.4)	29.2 (28.4-30.1)	28.6 (27.7-29.4)	27.6 (26.8-28.5)	<.0001	5590	-4.18	-5.44	-2.89
trunk fat mass (kg)^d^	9.3 (8.6-10.0)	9.1 (8.5-9.9)	8.8 (8.1-9.5)	8.3 (7.6-8.9)	<.0001	5590	-1.20	-1.61	-0.79
% trunk fat	24.8 (23.7-25.9)	24.7 (23.6-25.9)	23.9 (22.8-25.0)	22.8 (21.8-23.9)	<.0001	5590	-3.47	-4.79	-2.15
leg fat mass (kg)^d^	8.2 (7.9-8.5)	8.1 (7.8-8.4)	7.9 (7.6-8.2)	7.6 (7.4-7.9)	<.0001	5590	-2.09	-2.81	-1.37
% leg fat	36.3 (35.7-37.0)	35.9 (35.3-36.6)	35.4 (34.8-36.0)	34.7 (34.1-35.3)	<.0001	5590	-4.71	-5.91	-3.49
TLR	1.04 (1.00-1.07)	1.05 (1.01-1.08)	1.02 (0.99-1.06)	1.00 (0.96-1.04)	0.001	5590	-0.67	-1.12	-0.21

Abbreviations: PNNS-GS, Programme National Nutrition Santé Guideline Score; Q, quartile; 95% CI, 95% confidence intervalWC, waist circumference; HC, height circumference; WHR, waist to hip ratio; WHtR, waist to height ratio; TLR, trunk to leg ratio.

a Values are geometric means (95% confidence interval) adjusted for age, energy intake, tobacco smoking, current diet practice, season of dietary data collection, occupational status, educational level.

b P-value for linear trend across quartiles using contrast test

c Multivariate linear regression models provide regression coefficients (β) for the difference in z-score of log-transformed variables for each 1 point increase in PNNS-GS. We used exponentiation so that each coefficient is interpreted as the percent change of the expected z-score of variable for the increase of 1 point in PNNS-GS.

d Further adjusted for height.

### PNNS-GS and risk of the MetS

The likelihood of having MetS decreased across quartiles and for a 1-point increase of PNNS-GS (Table 5). In men, the lower likelihood of having MetS per 1-point increase in PNNS-GS was stronger than in women. In the whole sample and in men after adjustment for BMI (Model 2), ORs in Q2 were lower than in Q3. In addition, the ORs were closer to 1.00 although they remained significant. In women, OR for a 1-point increase of PNNS-GS was found non-significant in the model adjusted for BMI, however the trend was significant across quartiles and ORs for Q2 vs Q1 and Q3 vs Q1 were significantly lower than 1.

**Table 5:** Likelihood of having the Metabolic Syndrome according to adherence to nutritional guidelines (PNNS-GS) among French adults, NutriNet-Santé study, France, 2012.

	**Q1**	**Q2^a^**			**Q3 ^a^**			**Q4 ^a^**				**Continuous ^b^**			
		**OR**	**95% CI**		**OR**	**95% CI**		**OR**	**95% CI**		***P*^c^**	**OR**	**95% CI**		***P*^d^**
**Total n=7902**															
Model 1 ^e^	ref	0.73	0.59	0.90	0.72	0.59	0.89	0.59	0.48	0.73	<.0001	0.91	0.87	0.94	<.0001
Model 2 ^f^	ref	0.75	0.59	0.94	0.78	0.62	0.98	0.71	0.56	0.89	0.0170	0.94	0.90	0.98	0.007
**Men n=2264**															
Model 1 ^e^	ref	0.66	0.48	0.90	0.77	0.56	1.06	0.43	0.30	0.60	<.0001	0.86	0.80	0.91	<.0001
Model 2 ^f^	ref	0.78	0.55	1.11	1.02	0.72	1.44	0.55	0.38	0.80	0.003	0.90	0.84	0.97	0.01
**Women n=5638**															
Model 1 ^e^	ref	0.79	0.60	1.04	0.69	0.52	0.91	0.72	0.55	0.94	0.05	0.94	0.89	0.99	0.02
Model 2 ^f^	ref	0.73	0.54	0.99	0.65	0.48	0.89	0.81	0.60	1.10	0.04	0.97	0.91	1.03	0.30

Abbreviations: Q, Quartile; OR, Odds Ratio; 95% CI, 95% Confidence Interval

a Quartiles of PNNS-GS. Reference is the bottom quartile Q1.

b OR for the increase of 1 point of PNNS-GS

c P-value for trend across quartiles of PNNs-GS

d P-value for the effect of PNNS-GS taken as a continuous variable

e Model 1: Adjusted for gender (except for gender specific models), age, energy intake, time lag between dietary data collection and clinical visit, tobacco smoking, current diet practice, season of completion of 24h dietary record, educational level, occupational status.

f Model 2: Model 1 + BMI

As shown in Table S3, the negative association was mainly driven by 5 components of the PNNS-GS: fruits and vegetables, whole grain products, alcohol moderation, salt moderation and physical activity.

### Sensitivity analyses for risk of MetS

Two sets of supplementary analyses were carried out to test the robustness of the primary findings. Results are reported in Table S4. First, participants reporting being on a weight loss diet at the inception were excluded (n=3584). Second, participants on medication (antihypertensive, antidiabetic, treatment for hypercholesterolemia and treatment for hypertriglyceridemia) and those who had or have had a chronic cardiovascular disease (heart failure, stable angina, myocardial infarction and stroke) were excluded (n=1437).

Globally, the findings were similar in terms of direction and magnitude despite a loss of power due to sample size reduction leading to some associations which were not statistically significant.

## Discussion

In the present cross-sectional analysis, a better adherence to the French dietary guidelines was negatively associated with the MetS prevalence, with some individual MetS-related traits and with all markers of regional adiposity. These results support a potential beneficial role of official nutritional recommendations disseminated by public health authorities on cardiovascular health status even if inference for causality and temporality cannot be determined. Whereas SBP, DBP, triglycerides and WC were negatively associated with the PNNS-GS, some traits of the MetS, namely fasting blood glucose and serum HDL cholesterol concentrations, were not individually associated with the PNNS-GS.

The present analyses on a French sample of greater size than in the French Nutrition and Health Study (ENNS) study performed on a representative sample of the French population, allowed us to observe significant associations of PNNS-GS with the MetS. In ENNS, an inverse association was found significant only among young adults (<50y) not taking any medication [16]. No interaction between PNNS-GS and age was found in our study. On older participants of the SU. VI. MAX cohort (aged 45y and older at baseline), the PNNS-GS was associated with a 9% reduced risk of developing the MetS after 6 years of follow-up. However, after adjusting for baseline and change in BMI, this association remained significant only for severe MetS (waist criteria +3 or 4 criteria met) [17]. Some studies used other a priori scores in relation with the MetS, such as the HEI-2005 [12] or the DGAI [10,11]. In the US, among 18988 subjects of the NHANES [12], ORs of having the MetS per quartile of HEI-2005 were similar to our results (Q4 vs Q1, OR=0.65 (0.52, 0.82) where we found 0.71 (0.56-0.89)). The ORs in our study were somewhat stronger than those observed with the DGAI in Tehranian adults (OR Q4 vs Q1 = 0.79 (0.63-0.92) [11] or in the Frammingham Offspring Study where the negative association was found not significant (OR Q5 vs Q1= 0.81 (0.63, 1.04) [10].

A lower BP with increasing PNNS-GS is in line with the additive effects obtained from a diet rich in fruits and vegetables (such as the DASH diet) and a limitation in sodium intake [37].

Serum triglycerides concentrations are mostly influenced by alcohol and carbohydrates intakes [38,39], as confirmed by the analysis of separate components (Table S2). These dietary characteristics are well accounted for in the PNNS-GS computation (3 moderation components: alcohol consumption, sugar sweetened beverages, added sugars) and the results with the global PNNS-GS are particularly salient.

We did not observe any association between blood glucose and diet quality, as Nicklas et al. found with HEI-2005 in the study on NHANES [12], which is inconsistent with a recent review that showed cross-sectional association of a healthy diet with lower blood glucose [40]. Several arguments can be advanced to explain such discrepancies: the French PNNS recommends eating starchy foods at each meal according to appetite, regardless of their glycaemic index which can impact blood glucose [41], and consumption of 3 dairy products per day (4 for older people), whose effect on blood glucose is unclear [42,43]. Also, as shown by the low number of obese subjects and the very low number of participants under anti-diabetic drug, this might have led to a lack of power to detect an association with blood glucose.

Finally, no significant association of PNNS-GS with serum HDL–cholesterol concentrations was found. However, the components “physical activity”, “sugar-sweetened beverages” and “added sugars” were found to be positively significantly associated with serum HDL cholesterol concentrations (Table S2), which is coherent with existing evidence [44,45]. On the other hand, alcohol moderation (abstainer or moderate drinking) was negatively associated with serum HDL–cholesterol concentrations, which is not surprising [46] but might explain the overall non-significant association with PNNS-GS.

A great originality of our results is the data on adiposity status, not only assessed by BMI and WC, but also by % body fat, % trunk fat and TLR. Hence, to our knowledge it is the first study to focus on the relationship between adherence to nutritional guidelines and direct measures of adiposity in a large and diverse sample. We showed a firm decrease in any adiposity marker when PNNS-GS increases. One study in reproductive aged women found similar results on body fat composition assessed by DXA with adherence to a Mediterranean diet (aMED score) [19]. This is in agreement with other studies where adiposity was classically assessed either by BMI or WC, and diet quality referred to a Mediterranean diet [9,47] or dietary guidelines [9,10,12,47-50]. Abdominal adiposity is a key factor of the MetS since accumulation of visceral fat is associated with insulin resistance, inflammation and oxidative stress, which further contribute to the other components of the MetS [49].

After adjusting for BMI, the association between PNNS-GS and MetS among women was not significant, which suggests that the association was strongly driven by the adiposity component. Also, some factors such as nature of hormonal treatment (contraceptive or hormonal replacement therapies), or genetics that can influence the MetS were not taken into account here, hence residual confounding may remain.

The main strength of our study is the accurate measured biological and clinical data on a relatively large sample of French adults from the general population. Data on body composition provide new insights compared with usual measures of adiposity in cohort studies. Even if bio-impedance analysis is not the reference method to measure body fat composition, good validity indicators of TANITA compared with DXA were observed [51] and precise data on body composition is seldom available on such a large sample. Dietary intake was estimated through three 24HR which are known to provide accurate estimates of individual intake [52] while partly limiting measurement error as compared with food frequency questionnaire [53]. In addition, the web-based tool has shown high agreement with the reference method (interview with a dietician) [21]. Whereas dietary nutrients are likely to be interactive or synergistic in the food matrix [6], which can make it difficult to examine their separate effects on health outcomes, the use of an a priori score partially accounts for the complexity of the diet to detect the effect of the overall dietary behaviour. A priori score show limits due to the arbitrary choice of components and scoring system, hence they are not meant to describe dietary behaviors in an exhaustive way [54]. However, some items taken separately were not associated with the MetS whereas the global PNNS-GS was, illustrating the usefulness to consider diet as a whole when some separate effects cannot be detected [6].

The first and major limitation is the use of a cross-sectional design, which does not allow drawing conclusions on causal inference, but we adjusted for current diet practice and taking medication, which may limit the probability of reverse causality. Furthermore, reverse causality would lead to the absence of effect or a positive association, e.g. someone who is aware that they have impaired blood lipid levels would pay more attention to their diet and have a better PNNS-GS. Despite this, significant negative associations were found between PNNS-GS and a number of CVRF. Another limitation lies in the non-generalizability of our results as a selection bias might have occurred leading to a potential underestimation of the strength of the associations: participants in the NutriNet-Santé study are volunteers likely to be health-conscious and subjects included in the present analysis may be more healthy since they exhibited different socioeconomic and lifestyle characteristics from the total cohort. However, the percentages of subjects with the MetS and taking specific medication are only slightly lower than in the ENNS study (12.2% in our study vs 14% in ENNS), which is representative of the general population, although it is partly explained by the higher age of our population study (51y vs 45y in ENNS) [16].

In conclusion, the present cross-sectional study provides new insight on a larger sample of French adults and original data on body composition. The negative association between a higher adherence to the French dietary guidelines and a number of CVRF, the MetS prevalence and regional adiposity supports the importance of promoting the PNNS dietary guidelines in the population for the prevention of cardiometabolic abnormalities and hence, cardiovascular diseases. More studies, especially longitudinal studies, are needed to confirm these findings.

## Supporting Information

Table S1 **PNNS-GS: components and scores according to PNNS recommendations.**
(DOC)

Table S2 **Multivariate linear regression coefficients of CVRF associated with each component of the PNNS-GS, NutriNet-Santé study, France, 2012.**
(DOC)

Table S3 **Likelihood of having the Metabolic Syndrome according to components of the PNNS-GS among French adults, NutriNet-Santé study, France, 2012.**
(DOC)

Table S4 **Likelihood of having the MetS according to adherence to nutritional guidelines (PNNS-GS), sensitivity analyses on subsamples.**
(DOCX)
