# Supplementary material for: Association between Adherence to Nutritional Guidelines, the Metabolic Syndrome and Adiposity Markers in a French Adult General Population
Source: PLoS One. 2013 Oct 4;8(10):e76349. doi: 10.1371/journal.pone.0076349 (PMC3790685; doi:10.1371/journal.pone.0076349)
Supplement: Table S1 — PNNS-GS: components and scores according to PNNS recommendations. (DOC) [file pone.0076349.s001.doc]

Table S1. PNNS-GS: components and scores according to PNNS recommendations

|  | Recommendation | Scoring criteria^1^ | Score |
| --- | --- | --- | --- |
| **1.Fruits and vegetables** | At least 5/d | [0-3.5[ | 0 |
|  |  | [3.5-5[ | 0.5 |
|  |  | [5-7.5[ | 1 |
|  |  | ≥7.5 | 2 |
| **2.Bread, cereals, potatoes and legumes** | At each meal according to appetite | [0-1[ | 0 |
|  |  | [1-3[ | 0.5 |
|  |  | [3-6[ | 1 |
|  |  | ≥6 | 0.5 |
| ***3.Whole grain food*** | Choose whole grains and whole-grain breads more often | [0-1/3[ | 0 |
|  |  | [1/3-2/3[ | 0.5 |
|  |  | ≥2/3 | 1 |
| **4.Milk and dairy products** | 3/d (≥55-years-old: 3 to 4/d) | [0-1[ | 0 |
|  |  | [1-2.5[ | 0.5 |
|  |  | [2.5-3.5] (55-years-old: [2.5-4.5]) | 1 |
|  |  | >3.5 (55-years-old : >4.5) | 0 |
| **5.Meat, poultry seafood and eggs** | 1 to 2/d | 0 | 0 |
|  |  | ]0-1[ | 0.5 |
|  |  | [1-2] | 1 |
|  |  | >2 | 0.5 |
| ***6.Seafood*** | At least 2/week | < 2/week | 0 |
|  |  | ≥ 2/week | 1 |
| **7.Added fat** | Limit consumption | Lipids from added fat >16% EI^3^/d | 0 |
|  |  | Lipids from added fat ≤16% EI/d | 1 |
| ***8.Vegetable added fat*** | Favor fat of vegetable origin | No use of vegetable oil or ratio vegetable oil/total added fats≤0.5 | 0 |
|  |  | No use of added fats or ratio vegetable oil/total added fats >0.5 | 1 |
| **9.Sweetened foods** | Limit consumption | Added sugar from sweetened foods ≥17.5% EI/d | -0.5 |
|  |  | Added sugar from sweetened foods 17.5-12.5% EI/d | 0 |
|  |  | Added sugar from sweetened foods <12.5% EI/d | 1 |
| **Beverages** |  |  |  |
| ***10.Non-alcoholic beverages*** | Drink water as desired  Limit sweetened beverages: no more than 1 glass/d | <1l water and >250 ml soda/d | 0 |
|  |  | ≥1l water and >250 ml soda/d | 0.5 |
|  |  | <1l water and ≤250 ml soda/d | 0.75 |
|  |  | ≥1l water and ≤250 ml soda/d | 1 |
| ***11.Alcohol*** | Women advised to drink ≤2 glasses of wine/d and ≤3 glasses/d for men | Ethanol >20 g/d for women and >30g/d for men | 0 |
|  |  | Ethanol ≤20 g/d for women and ≤30g/d for men | 0.8 |
|  |  | Abstainers and irregular consumers (<once a week) | 1 |
| **12.Salt^2^** | Limit consumption | >12g/d | -0.5 |
|  |  | ]10-12] g/d | 0 |
|  |  | ]8-10] g/d | 0.5 |
|  |  | ]6-8] g/d | 1 |
|  |  | ≤6 g/d | 1.5 |
| **13.Physical activity** | At least the equivalent of 30 min/d of brisk walking | [0-30[ min/d | 0 |
|  |  | [30-60[ min/d | 1 |
|  |  | ≥60 min/d | 1.5 |
| Penalty on energy intake: |  | EI/EE≤1.05 | 0 |
| Ex: EI/EE=1.10 🡪 Total score reduced by 10%. |  | EI/EE>1.05 🡪 X=EI/EE-1 | Total score –X% |

^1^Servings per day unless otherwise indicated

^2^ Established according to French recommended dietary allowances

^3^ EI: energy intake without alcohol
