# Supplementary material for: Association between Adherence to Nutritional Guidelines, the Metabolic Syndrome and Adiposity Markers in a French Adult General Population
Source: PLoS One. 2013 Oct 4;8(10):e76349. doi: 10.1371/journal.pone.0076349 (PMC3790685; doi:10.1371/journal.pone.0076349)
Supplement: Table S2 — Multivariate linear regression coefficients of CVRF associated with each component of the PNNS-GS, NutriNet-Santé study, France, 2012. (DOC) [file pone.0076349.s002.doc]

**Table S2.** Multivariate linear regression coefficients of CVRF associated with each component of the PNNS-GS, NutriNet-Santé study, France, 2012

|  |  | β % ^a^ | 95% CI | | *P* |
| --- | --- | --- | --- | --- | --- |
| **SBP** | Fruits and vegetables≥5/d | -4.6 | -8.9 | -0.4 | 0.03 |
|  | Whole grains≥1/2 grain products | -6.5 | -11.5 | -1.8 | 0.01 |
|  | Starchy foods at each meal | -1.9 | -6.1 | 2.1 | 0.36 |
|  | Dairy products 3/day | 2.2 | -1.9 | 6.1 | 0.29 |
|  | Meat, fish and seafood, eggs 1 to 2/day | -0.8 | -4.7 | 2.9 | 0.68 |
|  | Fish and seafood 2/week | -0.3 | -4.4 | 3.6 | 0.87 |
|  | Alcohol moderation | -11.7 | -18.6 | -5.1 | 0.0004 |
|  | Limited added sugars | -2.3 | -6.5 | 1.8 | 0.28 |
|  | Limit sugar sweetened beverages <1/day | -7.6 | -12.4 | -3.0 | 0.001 |
|  | Limit salt consumption | 0.5 | -4.0 | 4.9 | 0.81 |
|  | Physical activity ≥30min/day | 4.2 | -0.4 | 8.6 | 0.07 |
|  | Limited added fat | -1.4 | -6.6 | 3.5 | 0.58 |
|  | Favour vegetal fat vs animal fat | -1.0 | -5.3 | 3.2 | 0.66 |
| **DBP** | Fruits and vegetables≥5/d | -6.3 | -11.2 | -1.7 | 0.01 |
|  | Whole grains≥1/2 grain products | -3.7 | -9.1 | 1.4 | 0.16 |
|  | Starchy foods at each meal | -0.3 | -4.9 | 4.1 | 0.89 |
|  | Dairy products 3/day | -1.7 | -6.4 | 2.8 | 0.47 |
|  | Meat, fish and seafood, eggs 1 to 2/day | -1.1 | -5.4 | 3.0 | 0.61 |
|  | Fish and seafood 2/week | -1.7 | -6.3 | 2.7 | 0.45 |
|  | Alcohol moderation | -5.1 | -12.4 | 1.7 | 0.14 |
|  | Limited added sugars | 4.0 | -0.4 | 8.2 | 0.07 |
|  | Limit sugar sweetened beverages <1/day | -5.7 | -10.9 | -0.7 | 0.03 |
|  | Limit salt consumption | -2.1 | -7.3 | 2.7 | 0.40 |
|  | Physical activity ≥30min/day | -3.4 | -8.9 | 1.8 | 0.20 |
|  | Limited added fat | -0.2 | -5.8 | 5.2 | 0.95 |
|  | Favour vegetal fat vs animal fat | -1.5 | -6.4 | 3.1 | 0.52 |
| **HDL-cholesterol** | Fruits and vegetables≥5/d | -1.9 | -6.3 | 2.2 | 0.37 |
|  | Whole grains≥1/2 grain products | 4.4 | -0.2 | 8.8 | 0.06 |
|  | Starchy foods at each meal | -3.7 | -8.1 | 0.5 | 0.09 |
|  | Dairy products 3/day | -0.3 | -4.6 | 3.9 | 0.91 |
|  | Meat, fish and seafood, eggs 1 to 2/day | 1.2 | -2.7 | 5.0 | 0.53 |
|  | Fish and seafood 2/week | 4.8 | 0.8 | 8.6 | 0.02 |
|  | Alcohol moderation | -32.0 | -40.5 | -24.1 | <.0001 |
|  | Limited added sugars | 5.8 | 1.8 | 9.7 | 0.005 |
|  | Limit sugar sweetened beverages <1/day | 6.8 | 2.5 | 11.0 | 0.002 |
|  | Limit salt consumption | -2.3 | -7.0 | 2.3 | 0.34 |
|  | Physical activity ≥30min/day | 12.3 | 8.0 | 16.5 | <.0001 |
|  | Limited added fat | -4.8 | -10.3 | 0.4 | 0.07 |
|  | Favour vegetal fat vs animal fat | -6.0 | -10.7 | -1.5 | 0.01 |
| **LDL-cholesterol** | Fruits and vegetables≥5/d | -3.6 | -8.3 | 1.0 | 0.12 |
|  | Whole grains≥1/2 grain products | 0.0 | -5.2 | 5.0 | 0.99 |
|  | Starchy foods at each meal | -2.9 | -7.6 | 1.6 | 0.21 |
|  | Dairy products 3/day | -4.0 | -8.8 | 0.6 | 0.09 |
|  | Meat, fish and seafood, eggs 1 to 2/day | 3.7 | -0.4 | 7.6 | 0.08 |
|  | Fish and seafood 2/week | 5.2 | 1.0 | 9.3 | 0.02 |
|  | Alcohol moderation | 3.9 | -2.8 | 10.1 | 0.25 |
|  | Limited added sugars | 1.1 | -3.4 | 5.5 | 0.62 |
|  | Limit sugar sweetened beverages <1/day | -2.4 | -7.4 | 2.5 | 0.34 |
|  | Limit salt consumption | 2.6 | -2.3 | 7.2 | 0.30 |
|  | Physical activity ≥30min/day | 2.9 | -2.3 | 7.8 | 0.27 |
|  | Limited added fat | 0.0 | -5.7 | 5.3 | 0.99 |
|  | Favour vegetal fat vs animal fat | -2.0 | -6.9 | 2.7 | 0.40 |
| **Serum triglycerides** | Fruits and vegetables≥5/d | -3.8 | -8.4 | 0.6 | 0.09 |
|  | Whole grains≥1/2 grain products | -3.2 | -8.4 | 1.7 | 0.20 |
|  | Starchy foods at each meal | 4.8 | 0.6 | 8.8 | 0.03 |
|  | Dairy products 3/day | -4.8 | -9.5 | -0.3 | 0.04 |
|  | Meat, fish and seafood, eggs 1 to 2/day | -1.1 | -5.3 | 2.9 | 0.59 |
|  | Fish and seafood 2/week | -3.7 | -8.3 | 0.6 | 0.10 |
|  | Alcohol moderation | 8.4 | 2.3 | 14.2 | 0.01 |
|  | Limited added sugars | -8.8 | -13.6 | -4.1 | 0.0002 |
|  | Limit sugar sweetened beverages <1/day | -8.3 | -13.5 | -3.3 | 0.001 |
|  | Limit salt consumption | -1.4 | -6.4 | 3.3 | 0.57 |
|  | Physical activity ≥30min/day | -7.9 | -13.5 | -2.6 | 0.003 |
|  | Limited added fat | 2.2 | -3.2 | 7.3 | 0.42 |
|  | Favour vegetal fat vs animal fat | -0.4 | -5.1 | 4.1 | 0.87 |
| **Blood glucose** | Fruits and vegetables≥5/d | -4.2 | -8.8 | 0.1 | 0.06 |
|  | Whole grains≥1/2 grain products | -1.4 | -6.4 | 3.3 | 0.57 |
|  | Starchy foods at each meal | -4.3 | -8.8 | 0.0 | 0.05 |
|  | Dairy products 3/day | 4.1 | -0.2 | 8.1 | 0.06 |
|  | Meat, fish and seafood, eggs 1 to 2/day | 0.5 | -3.5 | 4.4 | 0.79 |
|  | Fish and seafood 2/week | 4.2 | 0.1 | 8.1 | 0.05 |
|  | Alcohol moderation | -17.6 | -25.3 | -10.3 | <.0001 |
|  | Limited added sugars | 1.9 | -2.4 | 6.0 | 0.38 |
|  | Limit sugar sweetened beverages <1/day | -2.0 | -6.8 | 2.6 | 0.40 |
|  | Limit salt consumption | 0.3 | -4.5 | 4.9 | 0.90 |
|  | Physical activity ≥30min/day | -0.2 | -5.3 | 4.6 | 0.93 |
|  | Limited added fat | -2.5 | -8.0 | 2.7 | 0.35 |
|  | Favour vegetal fat vs animal fat | 1.6 | -2.9 | 5.9 | 0.49 |
| **WC** | Fruits and vegetables≥5/d | -2.9 | -5.1 | -0.8 | 0.01 |
|  | Whole grains≥1/2 grain products | -4.4 | -6.9 | -2.0 | 0.0003 |
|  | Starchy foods at each meal | 0.8 | -1.3 | 2.8 | 0.47 |
|  | Dairy products 3/day | -0.4 | -2.5 | 1.7 | 0.72 |
|  | Meat, fish and seafood, eggs 1 to 2/day | 0.6 | -1.4 | 2.5 | 0.58 |
|  | Fish and seafood 2/week | -1.7 | -3.8 | 0.4 | 0.12 |
|  | Alcohol moderation | -4.5 | -7.8 | -1.2 | 0.01 |
|  | Limited added sugars | -2.4 | -4.6 | -0.3 | 0.02 |
|  | Limit sugar sweetened beverages <1/day | -1.1 | -3.4 | 1.1 | 0.33 |
|  | Limit salt consumption | -3.5 | -5.9 | -1.2 | 0.003 |
|  | Physical activity ≥30min/day | -5.5 | -8.1 | -3.0 | <.0001 |
|  | Limited added fat | -0.9 | -3.5 | 1.7 | 0.52 |
|  | Favour vegetal fat vs animal fat | -0.3 | -2.5 | 1.9 | 0.82 |

^a^ Multivariate linear regression models provide regression coefficients (β) for the difference in z-score of log-transformed variables when the recommendation is attained vs not attained. We used exponentiation so that each coefficient is interpreted as the percent change of the expected z-score when the recommendation is met vs not.

Models were adjusted for age, gender, tobacco smoking, current diet practice, season of completion of 24h dietary record, educational level, occupational status, PNNS-GS minus the corresponding component, treatment for the specific outcome and BMI (except for WC).
