# Supplementary material for: Association between Adherence to Nutritional Guidelines, the Metabolic Syndrome and Adiposity Markers in a French Adult General Population
Source: PLoS One. 2013 Oct 4;8(10):e76349. doi: 10.1371/journal.pone.0076349 (PMC3790685; doi:10.1371/journal.pone.0076349)
Supplement: Table S3 — Likelihood of having the Metabolic Syndrome according to components of the PNNS-GS among French adults, NutriNet-Santé study, France, 2012. (DOC) [file pone.0076349.s003.doc]

**Table S3**. Likelihood of having the Metabolic Syndrome according to components of the PNNS-GS among French adults, NutriNet-Santé study, France, 2012

|  |  | **OR^a^** | **95% CI** | |
| --- | --- | --- | --- | --- |
| Fruits and vegetables≥5/d | Model 1 ^b^ | **0.77** | **0.67** | **0.89** |
|  | Model 2 ^c^ | **0.80** | **0.69** | **0.93** |
|  | Model 2+BMI | **0.82** | **0.70** | **0.97** |
| Starchy foods at each meal | Model 1 ^b^ | 0.93 | 0.80 | 1.08 |
|  | Model 2 ^c^ | 1.00 | 0.86 | 1.17 |
|  | Model 2+BMI | 0.97 | 0.82 | 1.14 |
| Whole grains≥1/2 grain products | Model 1 ^b^ | **0.73** | **0.61** | **0.86** |
|  | Model 2 ^c^ | **0.76** | **0.64** | **0.90** |
|  | Model 2+BMI | 0.87 | 0.72 | 1.05 |
| Dairy products 3/day | Model 1 ^b^ | 1.13 | 0.98 | 1.31 |
|  | Model 2 ^c^ | 1.13 | 0.97 | 1.31 |
|  | Model 2+BMI | 1.03 | 0.88 | 1.22 |
| Meat. fish and seafood. eggs 1 to 2/day | Model 1 ^b^ | 1.03 | 0.90 | 1.19 |
|  | Model 2 ^c^ | 1.06 | 0.92 | 1.22 |
|  | Model 2+BMI | 1.07 | 0.91 | 1.25 |
| Fish and seafood 2/week | Model 1 ^b^ | 0.90 | 0.77 | 1.04 |
|  | Model 2 ^c^ | 0.93 | 0.80 | 1.08 |
|  | Model 2+BMI | 0.99 | 0.84 | 1.17 |
| Limit sugar sweetened beverages <1/day | Model 1 ^b^ | **0.85** | **0.73** | **0.98** |
|  | Model 2 ^c^ | **0.83** | **0.71** | **0.96** |
|  | Model 2+BMI | **0.82** | **0.70** | **0.97** |
| Alcohol moderation | Model 1 ^b^ | **0.79** | **0.64** | **0.96** |
|  | Model 2 ^c^ | 0.86 | 0.70 | 1.05 |
|  | Model 2+BMI | 0.85 | 0.68 | 1.06 |
| Limited added sugars | Model 1 ^b^ | 1.25 | 1.05 | 1.48 |
|  | Model 2 ^c^ | 1.17 | 0.98 | 1.40 |
|  | Model 2+BMI | 0.96 | 0.79 | 1.16 |
| Limited added fat | Model 1 ^b^ | 1.11 | 0.92 | 1.33 |
|  | Model 2 ^c^ | 1.09 | 0.90 | 1.32 |
|  | Model 2+BMI | 1.08 | 0.87 | 1.32 |
| Favour vegetal fat vs animal fat | Model 1 ^b^ | 1.04 | 0.89 | 1.22 |
|  | Model 2 ^c^ | 1.03 | 0.87 | 1.20 |
|  | Model 2+BMI | 1.10 | 0.92 | 1.31 |
| Limit salt consumption | Model 1 ^b^ | **0.68** | **0.58** | **0.80** |
|  | Model 2 ^c^ | **0.67** | **0.56** | **0.79** |
|  | Model 2+BMI | 0.86 | 0.71 | 1.03 |
| Physical activity ≥30min/day | Model 1 ^b^ | **0.61** | **0.52** | **0.72** |
|  | Model 2 ^c^ | **0.65** | **0.54** | **0.77** |
|  | Model 2+BMI | **0.82** | **0.68** | **1.00** |

^a^ OR for compliance with the recommendation (score ≥1 for the component vs <1)

^b^ Model 1: Adjusted for gender, age, energy intake and time lag between dietary data collection and clinical visit.

^c^ Model 2: Model 1 + tobacco smoking, current diet practice, season of completion of 24h dietary record, educational level, occupational status, and PNNS-GS minus the corresponding component
