# Supplementary material for: Association between Adherence to Nutritional Guidelines, the Metabolic Syndrome and Adiposity Markers in a French Adult General Population
Source: PLoS One. 2013 Oct 4;8(10):e76349. doi: 10.1371/journal.pone.0076349 (PMC3790685; doi:10.1371/journal.pone.0076349)
Supplement: Table S4 — Likelihood of having the MetS according to adherence to nutritional guidelines (PNNS-GS), sensitivity analyses on subsamples. (DOCX) [file pone.0076349.s004.docx]

**Table S4**. Likelihood of having the MetS according to adherence to nutritional guidelines (PNNS-GS), sensitivity analyses on subsamples.

|  | TOTAL |  | MEN |  | WOMEN |  |
| --- | --- | --- | --- | --- | --- | --- |
| **Total sample** | n=7902 |  | n=2264 |  | n=5638 |  |
| MetS (n, %) | 963 | 12.2 | 427 | 18.9 | 536 | 9.5 |
| Age (mean, SD) | 50.8 | 13.6 | 54.5 | 13.6 | 49.3 | 13.3 |
| On a weight loss diet (n, %) | 3584 | 45.4 | 567 | 25 | 3017 | 53.5 |
| Taking medication (n, %) | 1294 | 16.4 | 573 | 25.3 | 721 | 12.8 |
| Having chronic condition (n, %) | 305 | 3.9 | 183 | 8.1 | 122 | 2.2 |
| OR continuous (OR, 95%CI) ^a^ | | |  |  |  |  |
| Model 1^b^ | 0.91 | 0.87 - 0.94 | 0.86 | 0.80 - 0.91 | 0.94 | 0.89 - 0.99 |
| Model 2 ^c^ | 0.94 | 0.90 - 0.98 | 0.90 | 0.84 - 0.97 | 0.97 | 0.91 - 1.03 |
| **Sample 1 : exclusion of participants on a weight loss diet** | n=4318 | | n=1697 |  | n=2621 | |
| MetS (n, %) | 372 | 8.62 | 240 | 14.14 | 132 | 5.04 |
| Age (mean, SD) | 50.7 | 14.4 | 54.3 | 14.0 | 48.3 | 14.2 |
| PNNS-GS (mean, SD) | 9.2 | 2.0 | 9.3 | 1.9 | 9.2 | 2.0 |
| OR continuous (95%CI) ^a^ | | |  |  |  |  |
| Model 1^b^ | 0.89 | 0.83 - 0.94 | 0.84 | 0.78 - 0.91 | 0.94 | 0.85 - 1.04 |
| Model 2 ^c^ | 0.93 | 0.87 - 1.00 | 0.90 | 0.82 - 0.99 | 0.97 | 0.86 - 1.09 |
| **Sample 2 : exclusion of participants on medication or chronic CVD** | n=6465 | | n=1624 |  | n=4841 | |
| MetS (n, %) | 483 | 7.47 | 197 | 12.13 | 286 | 5.91 |
| PNNS-GS (mean, SD) | 9.3 | 2.0 | 9.2 | 1.9 | 9.4 | 2.0 |
| Age (mean, SD) | 48.6 | 13.6 | 51.3 | 14.1 | 47.6 | 13.3 |
| OR continuous (95%CI) ^a^ | | |  |  |  |  |
| Model 1^b^ | 0.91 | 0.86 - 0.96 | 0.88 | 0.80 - 0.96 | 0.92 | 0.86 - 0.99 |
| Model 2 ^c^ | 0.95 | 0.90 - 1.01 | 0.97 | 0.88 - 1.07 | 0.95 | 0.88 - 1.02 |

^a^ OR for a 1-point increase in PNNS-GS

^b^ Model 1: Adjusted for gender (except for gender specific models), age, energy intake, time lag between dietary data collection and clinical visit, tobacco smoking, current diet practice, season of completion of 24h dietary record, educational level, occupational status.

^c^ Model 2: Model 1 + BMI
